# Correlation between serum ferritin in early pregnancy and hypertensive disorders in pregnancy

**DOI:** 10.3389/fnut.2023.1151410

**Published:** 2023-04-17

**Authors:** Zhuanji Fang, Shuisen Zheng, Yi Xie, Shunhe Lin, Huale Zhang, Jianying Yan

**Affiliations:** ^1^Department of Obstetrics, Fujian Maternity and Child Health Hospital, Fuzhou, Fujian, China; ^2^College of Clinical Medicine for Obstetrics & Gynecology and Pediatrics, Fujian Medical University, Fuzhou, Fujian, China; ^3^Fujian Medical University, Fujian, China; ^4^Department of Gynecology, Fujian Maternity, and Child Health Hospital, Fuzhou, Fujian, China; ^5^Laboratory of Maternal-Fetal Medicine, Fujian Clinical Research Center for Maternal-Fetal Medicine, Fuzhou, Fujian, China

**Keywords:** early pregnancy, generalized additive model, hypertensive disorders of pregnancy, iron supplementation, serum ferritin

## Abstract

**Objective:**

To explore the correlation between serum ferritin (SF) in early pregnancy and the risk of hypertensive disorders in pregnancy (HDP).

**Method:**

A retrospective cohort study was conducted on 43,421 pregnant women with singleton pregnancies who underwent antenatal checkups at Fujian Provincial Maternal and Child Health Hospital from January 2018 to December 2020. Based on pregnancy records, women were classified as non-hypertensive, having gestational hypertension, preeclampsia and preeclampsia with severe features according to the degree of the disease. General baseline data, and SF levels in the early (up to 12 gestational weeks) and late (after 28  weeks of gestation) stages of pregnancy were collected. The significance of the characteristic variables was assessed using a random forest algorithm, and the correlation between early pregnancy SF levels and the incidence of HDP was further analyzed using logistics regression adjusted for confounders. A generalized additive model (GAM) was fitted to a smoothed graph of the relationship between early pregnancy SF levels and HDP, and a threshold effect analysis was performed to find the threshold values of early pregnancy SF for iron supplementation therapy.

**Result:**

A total of 30,703 pregnant women were included. There were 1,103 women who were diagnosed with HDP. Of them, 418 had gestational hypertension, 12 had chronic hypertension without SPE, 332 - preeclampsia and 341 women had preeclampsia with severe features. Levels of SF in early and late pregnancy were significantly higher (*p* < 0.001) in women with HDP compared to non-hypertensive women and the difference was more pronounced in early pregnancy. The random forest algorithm showed that early pregnancy SF was more effective in predicting HDP compared to late pregnancy SF levels and was also an independent risk factor for HDP (adjusted odds ratio (AOR) = 1.07, 95% CI [1.05,1.09]) after correction for confounding factors. Early pregnancy SF >64.22  mg/l was associated with higher risk of developing hypertensive disorders.

**Conclusion:**

Risk of pregnancy-related hypertensive disorders increases with increasing early pregnancy SF levels. SF levels may therefore be used to further develop guidelines for iron supplementation therapy in pregnant women.

## Introduction

Hypertensive disorders of pregnancy (HDP) including chronic hypertension, gestational hypertension, preeclampsia-eclampsia, and chronic hypertension with superimposed preeclampsia, are major contributors to maternal and fetal perinatal mortality and adverse outcomes (1). However, their pathophysiological mechanisms are still not clear. Some studies suggest that development of hypertensive disorders of pregnancy results from iron-mediated oxidative stress (2). Currently, prenatal iron supplementation is routinely recommended worldwide to prevent iron deficiency anemia (3). At the same time, studies show that excess iron may increase the risk of low birth weight (4), preterm births (5), and especially, hypertensive disorders in pregnancy (6). Therefore, most studies support lowering the dose of supplemented iron during pregnancy (7). There is still a lack of in-depth research on the threshold for iron supplementation and limited evidence-based research slows down the development of specific monitoring and interventions. Serum ferritin (SF) is a stable glycoprotein that is unaffected by recent iron intake and is one of the body’s primary forms of iron storage, making it the most effective indicator of iron status. However, ferritin is also an acute phase reactant and may be elevated in the context of acute inflammation (8). In preeclampsia (PE), oxidative stress, induced in the ischemic placenta causes a systemic inflammatory response and activates maternal endothelial cells (9). Recent studies suggests that increased ferritin concentrations are linked to a higher risk of PE (10) and are associated with a higher rate of unfavorable neonatal outcomes in women with PE.

Based on these observations, we hypothesized that there is a link between the levels of serum ferritin in early pregnancy and the development of pregnancy-related hypertensive disorders. The main goal of this study was to investigate this potential association, and to identify a threshold serum ferritin level for the prenatal iron supplementation therapy. These findings may allow to further develop guidelines of iron supplementation therapy during pregnancy and to improve pregnancy outcomes.

## Materials and methods

### Study subjects

**Figure 1:**
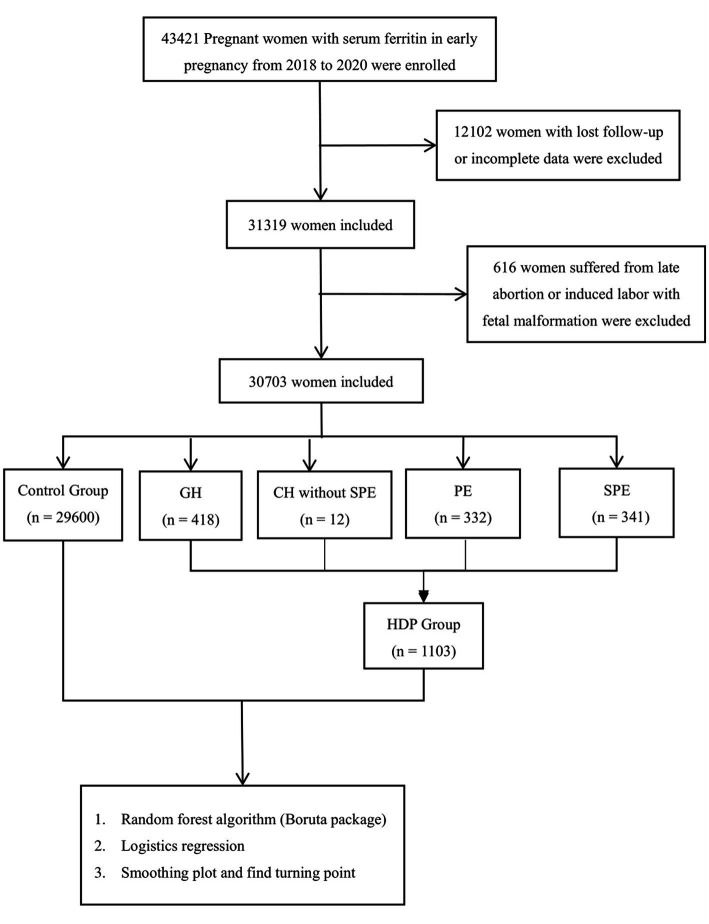
Flowchart of the enrolled pregnancy women.

Pregnant women who had their records established at Fujian Maternal and Child Health Hospital from January 2018 to December 2020, had regular maternity check-ups until delivery and had their SF tested at 11 weeks of pregnancy, were included in the cohort. A total of 30,703 pregnant women were selected and divided into the control group (29,600 cases), gestational hypertension group (418 cases), chronic hypertension without SPE (12 cases), preeclampsia group (332 cases) and preeclampsia with severe features group (341 cases). A flowchart, showing study design is shown in Figure 1.

### Diagnostic criteria

HDP: definition of HDP in this study included gestational hypertension, preeclampsia-eclampsia, chronic hypertension, chronic hypertension + preeclampsia, per guidelines for the diagnosis and treatment of HDP (2020) issued by the Chinese Medical Association Obstetrics and Gynecology Branch (11).

Diagnostic criteria of gestational hypertension: SBP ≥ 140 mmHg and/or DBP ≥90 mmHg on two occasions at least 4 h apart after 20 weeks of gestation in a previously normotensive patient; no evidence of proteinuria (<300 mg in 24 h).

Diagnostic criteria of preeclampsia: Blood pressure: 140 mm Hg or higher systolic or 90 mm Hg or higher diastolic after 20 weeks of gestation in a woman with previously normal blood pressure; proteinuria (≥300 mg in 24 h), or any one of the following organs or systems involved: heart, lung, liver, kidney and other vital organs, or abnormal changes in the blood system, digestive system, nervous system, placenta/fetus affected.

Chronic hypertension: blood pressure ≥140 mm Hg systolic and/or 90 mm Hg diastolic before 20 weeks of gestation, use of antihypertensive medications before pregnancy, or persistence of hypertension for >12 weeks after delivery.

### Research methodology

Clinical information was collected from hospital electronic medical records for both groups of pregnant women, including:

1. General information: age, education, height, pre-pregnancy body mass, delivery body mass and body mass index, number of births, number of pregnancies, number of deliveries, etc.
2. Maternal complications and comorbidities: hypertensive disorders in pregnancy, gestational diabetes, premature rupture of membranes, low lying placenta, hyperthyroidism, hypothyroidism, hepatitis B virus infection in pregnancy, etc.
3. Laboratory tests at diagnosis: alanine aminotransferase (ALT), aspartate aminotransaminase (AST), gamma-glutamyltransferase (GGT), lactate Lactate Dehydrogenase (LDH), Triglyceride (TG), Cholesterol (CHOL), White Blood Cell Count (WBC), Neutrophils (NE), Monocyte Count (MO), Lymphocytes (LY), hemoglobin (HGB), platelet count (PLT).
4. SF levels during early (before the end of the 12th week of gestation) and late pregnancy (gestational age greater than 28 weeks). Abbott A16200 automatic biochemical analyzer was used to detect Serum Ferritin (reference range: 4.63–204.0 ng/ml).

Follow-up: All women were followed up for 12 weeks postpartum to establish a definitive diagnosis of HDP and to monitor any potential incidence of postpartum preeclampsia.

### Statistical methods

The R software (V3.6.2) package was used for statistics. The measurement data were expressed as mean ± standard deviation (x ± s) and compared by t-test. Count data were expressed as numbers n or rates (%) and analyzed using the χ2 test, corrected chi-square test, or Fisher’s exact probability method. The significance of the characteristic variables in predicting hypertensive disorders in pregnancy was calculated using the random forest algorithm (Boruta package), and the correlation between early pregnancy SF levels and HDP was further explored using a logistic regression model corrected for confounders to adjust for the odds ratio (OR) and 95% confidence interval (CI). *p* < 0.05 was considered statistically significant. A smoothed graph of the SF levels-HDP relationship was fitted using the gam model, and a threshold effect analysis was further used to find the threshold of SF for iron supplementation therapy during pregnancy.

### Ethical approval

Ethical committee of the Fujian Maternity and Child Health Hospital approved this study (2020YJ183). As there were no interventions to the women’ care at any stage, the need for ethical approval and written informed consent was waived.

## Results

### Basic characteristics of the women

A total of 1,103 women (3.59% of the total number of women included in the study) were diagnosed with different types of HDP. and 54 of these had early-onset preeclampsia (5%). Patient baseline characteristics are shown in Table 1.

**Table 1:** General baseline information.

	Total (*n* = 30,703)	Control Group (*n* = 29,600)	HDR group (*n* = 1,103)	*p* value	GH/CH (*n* = 430)	PE (*n* = 332)	SPE (*n* = 341)	*P* value
Age at delivery (y)	28 (26, 32)	28 (26, 32)	30 (27, 34)	<0.001	29 (27, 33)	30 (27, 34)	30 (27, 34)	<0.001
Education level				0.985				0.969
Primary school or below [n (%)]	264 (1)	254 (1)	10 (1)		3 (1)	3 (1)	4 (1)	
Junior and Senior high school [n (%)]	8,554 (28)	8,247 (28)	307 (28)		117 (27)	98 (30)	92 (27)	
College or higher [n (%)]	21,885 (71)	21,099 (71)	786 (71)		310 (72)	231 (70)	245 (72)	
Gravity				0.017				0.125
G ≤ 2	20,950 (68)	20,154 (68)	796 (72)		316 (73)	241 (73)	239 (70)	
4 ≥ G>2	8,391 (27)	8,128(27)	263 (24)		96 (22)	77 (23)	90 (26)	
G ≥ 5	1,362 (4)	1,318 (4)	44(4)		18 (4)	14 (4)	12 (4)	
Parity n (%)				<0.001				<0.001
0	14,779 (48)	14,151 (48)	628 (57)		240 (56)	189 (57)	199 (58)	
1	14,757 (48)	14,311 (48)	446 (40)		176 (41)	135 (41)	135 (40)	
2	1,116 (4)	1,089 (4)	27 (2)		14 (3)	6 (2)	7 (2)	
3	47 (0)	45 (0)	2 (0)		0 (0)	2 (1)	0 (0)	
4	4 (0)	4 (0)	0 (0)		0 (0)	0 (0)	0 (0)	
Height(m)	1.6 (1.57, 1.63)	1.6 (1.57, 1.63)	1.6 (1.56, 1.63)	0.931	1.6 (1.57, 1.63)	1.6 (1.56, 1.64)	1.6 (1.56, 1.63)	0.178
Pre-pregnancy BMI (kg/m^2^)	20.32 (18.81, 22.19)	20.31 (18.78, 22.1)	21.56 (19.81, 23.92)	<0.001	21.51 (19.91, 23.73)	22.16 (20.32, 24.53)	20.96 (19.29, 23.44)	<0.001
Prenatal BMI (kg/m^2^)	25.96 (24.22, 27.89)	25.91 (24.17, 27.82)	27.59 (25.64, 29.76)	<0.001	27.39 (25.41, 29.38)	28.04 (26.02, 30.48)	27.39 (25.39, 29.67)	<0.001
Net weight gain per week (kg)	0.36 (0.29, 0.42)	0.36 (0.29, 0.42)	0.38 (0.3, 0.47)	<0.001	0.37 (0.3, 0.44)	0.38 (0.29, 0.47)	0.4 (0.32, 0.49)	<0.001
Gestational age (weeks)	39.43 (38.71, 40.29)	39.43 (38.71, 40.29)	39 (37.71, 40)	<0.001	39.43 (38.57, 40.29)	39.14 (38.14, 40)	38 (36, 39.43)	<0.001
GDM n (%)								<0.001
No	25,315 (82)	24,492 (83)	823 (75)	<0.001	325 (76)	241 (73)	257 (75)	
Yes	5,388 (18)	5,108 (17)	280 (25)		105 (24)	91 (27)	84 (25)	
HBV infection n (%)				0.648				0.62
No	27,189 (89)	26,207 (89)	982 (89)		388 (90)	296 (89)	298 (87)	
Yes	3,514 (11)	3,393 (11)	121 (11)		42 (10)	36 (11)	43 (13)	
Hyperthyroidism n (%)				0.030				0.003
No	30,167 (98)	29,093 (98)	1,074 (97)		422 (98)	326 (98)	326 (96)	
Yes	536 (2)	507 (2)	29 (3)		8 (2)	6 (2)	15 (4)	
Hypothyroidism n (%)				0.682				0.728
No	29,373 (96)	28,321 (96)	1,052 (95)		412 (96)	318 (96)	322 (94)	
Yes	1,330 (4)	1,279 (4)	51 (5)		18 (4)	14 (4)	19 (6)	
Uterine malformation n (%)				0.290				0.039
No	30,443 (99)	29,353 (99)	1,090 (99)		428 (100)	329 (99)	333 (98)	
Yes	260 (1)	247 (1)	13 (1)		2 (0)	3 (1)	8 (2)	
SGA n (%)				<0.001				<0.001
	29,830 (97)	28,831 (97)	999 (91)		412 (96)	321 (97)	266 (78)	
	873 (3)	769 (3)	104 (9)		18 (4)	11 (3)	75 (22)	
ALT (U/L)	12.2 (9, 17.7)	12.1 (9, 17.4)	13.8 (9.8, 20.55)	<0.001	13.4 (9.72, 18.7)	12.65 (9, 18)	16.4 (11.1, 27.6)	<0.001
AST (U/L)	17 (14, 21)	17 (14, 20.8)	18.9 (15, 24.1)	<0.001	18 (14.72, 22)	17.6 (14, 22.22)	21.5 (16.6, 31.2)	<0.001
rGGT (U/L)	11.6 (9.2, 16.2)	11.6 (9.2, 16)	12.7 (9.6, 19.5)	<0.001	12.15 (9.7, 18)	11.9 (9, 18.12)	14.3 (10, 25.8)	<0.001
ALB (g/L)	34.7 (33, 36.4)	34.8 (33, 36.4)	33.3 (31, 35.4)	<0.001	34.4 (32.7, 36.2)	33.25 (31, 35.5)	31.8 (29.7, 33.9)	<0.001
CHOL (mmol/L)	6.07 (5.36, 6.88)	6.08 (5.37, 6.89)	5.88 (5.07, 6.81)	<0.001	5.96 (5.26, 6.63)	5.86 (5.01, 6.82)	5.77 (4.92, 6.92)	<0.001
TG (mmol/L)	3.02 (2.38, 3.89)	3.01 (2.37, 3.87)	3.31 (2.65, 4.3)	<0.001	3.21 (2.62, 4.09)	3.42 (2.68, 4.54)	3.35 (2.66, 4.3)	<0.001
LDH (U/L)	180.8 (158.1, 234)	180.3 (157.8, 231.9)	199.2 (169.75, 308.2)	<0.001	189 (165.9, 244.68)	191.9 (166.85, 239.77)	238.6 (188.2, 394.6)	<0.001
WBC (×10^9^)	9.77 (8.16, 11.75)	9.75 (8.15, 11.73)	10.11 (8.37, 12.72)	<0.001	9.82 (8.3, 11.7)	10.31 (8.69, 12.87)	10.71 (8.35, 13.67)	<0.001
NE (×10^9^)	7.25 (5.84, 9.09)	7.25 (5.84, 9.06)	7.5 (5.88, 9.68)	0.002	7.3 (5.85, 9.38)	7.38 (5.89, 9.52)	7.91 (5.88, 10.31)	0.002
LY (×10^9^)	1.59 (1.32, 1.92)	1.59 (1.32, 1.92)	1.63 (1.33, 2.01)	0.008	1.66 (1.38, 1.97)	1.6 (1.32, 2.04)	1.58 (1.29, 2.07)	0.031
MO (×10^9^)	0.66 (0.53, 0.81)	0.66 (0.53, 0.81)	0.67 (0.53, 0.83)	0.22	0.66 (0.54, 0.82)	0.67 (0.54, 0.84)	0.66 (0.52, 0.83)	0.449
HGB (g/L)	119 (110, 127)	119 (110, 127)	119 (107, 127)	0.129	121 (112, 130)	117 (107, 126)	116 (102, 126)	<0.001
PLT (×10^9^)	207 (175, 243)	207 (175, 243)	206 (173, 244)	0.608	214 (180, 250)	207 (176, 245)	197 (159, 234)	<0.001
Serum ferritin in early (until gestational week12) pregnancy (ng/mL)	22.6 (14.38, 36.06)	22.43 (14.3, 35.8)	27.2 (16.6, 43.65)	<0.001	24.8 (15.02, 39.11)	27.67 (17.47, 44.73)	28.85 (18.35, 47.13)	<0.001
Serum ferritin in late pregnancy (gestational week greater than 28 weeks)(ng/mL)	24.2 (12.7, 50.56)	24.06 (12.65, 50.3)	29.2 (14.7, 61.02)	<0.001	27.85 (14.12, 54.13)	30.58 (15.37, 62)	29.39 (14.77, 69.3)	<0.001

HDP, Hypertension Disorders in Pregnancy; CH, chronic hypertension without SPE. BMI, Body mass index; HBV, Hepatitis B virus; GDM, Gestational diabetes mellitus; SGA, small for gestational age; ALT, Alanine aminotransaminase; AST, Aspartame aminotransferase; GGT, transglutaminase; ALB, Albumin; CHOL, Cholesterol; TG, Triglycerides; LDH, Lactate dehydrogenase; WBC, White blood cell count; NE, Neutrophil count; LY, Lymphocyte count; MO, Monocyte count; HGB, Hemoglobin; PLT, Platelet count.

No statistically significant differences were noted between the groups in terms of education level and height. Age at delivery was significantly higher in the HDP groups compared to control [30 (27, 34) and 28 (26, 32), respectively]. Similarly, pre-pregnancy BMI, prenatal BMI, average net weight gain per week were significantly higher in all HDR groups. There was a statistically significant differences in the gravidity and the gestational age between the groups (*p* < 0.05). In terms of parity, there was a significantly higher percentage of nulliparous women in the GH, PE and SPE groups compared to the control group, while the number of primiparous and multiparous women was higher in the control group (*p* < 0.001). In terms of complications, the differences in the occurrence of Hepatitis B virus (HBV) infection and hypothyroidism among the four groups were not significant, while the diagnosis of HDP correlated with higher occurrence of gestational diabetes mellites (GDM), hyperthyroidism and uterine malformation (*p* < 0.05). In terms of laboratory tests, the differences in MO between the four groups of pregnant women were not statistically significant. HDP groups had higher levels of ALT, GGT, AST, LDH, WBC, NE, TG, LY, HGB and PLT, and lower levels of ALB and CHOL (*p* < 0.05; Table 1). Levels of SF in early and late pregnancy were significantly higher (*p* < 0.001) in all HDP groups (Table 1), and this difference was more pronounced in early pregnancy.

### Predictors of HPD

**Figure 2:**
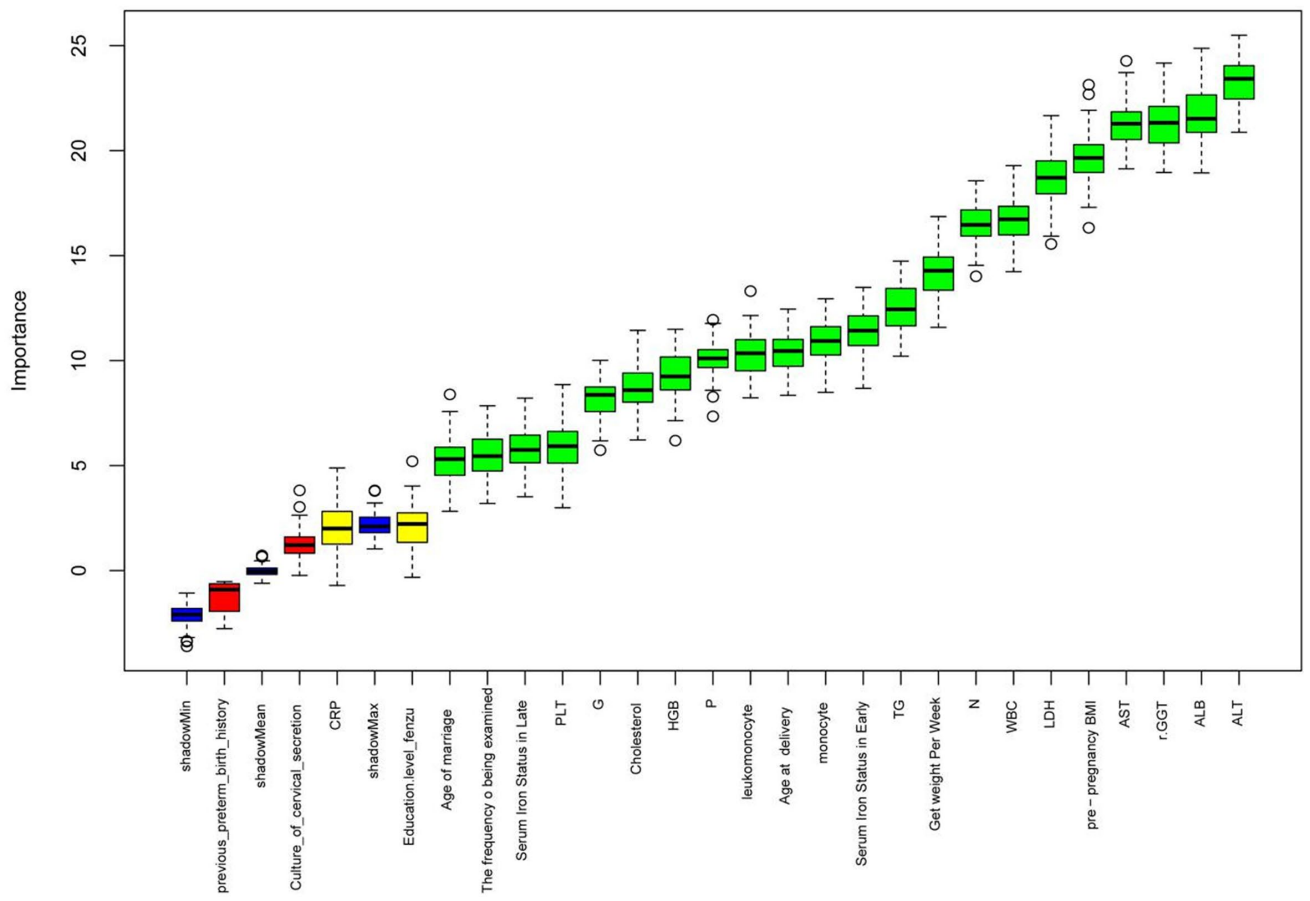
Schematic diagram for assessing the importance of variables. (The horizontal axis is the characteristic variable predicting hypertensive disorders in pregnancy, the vertical axis is the importance score, green is the important variable, red is the insignificant variable, blue is the shadow variable and yellow is the Tentative variable.)

The Boruta algorithm of random forest was used to assess the importance of the characteristic variables in predicting hypertensive disorders in pregnancy. The characteristic variables were ranked according to their importance scores (Figure 2). The top-ranked important predictor variables were ALT, ALB, rGGT, AST, pre-pregnancy BMI, LDH, N, net weight per week, TG, SF in early pregnancy and SF in late pregnancy; pregnancy SF levels in early were significantly more important than in late pregnancy.

**Figure 3:**
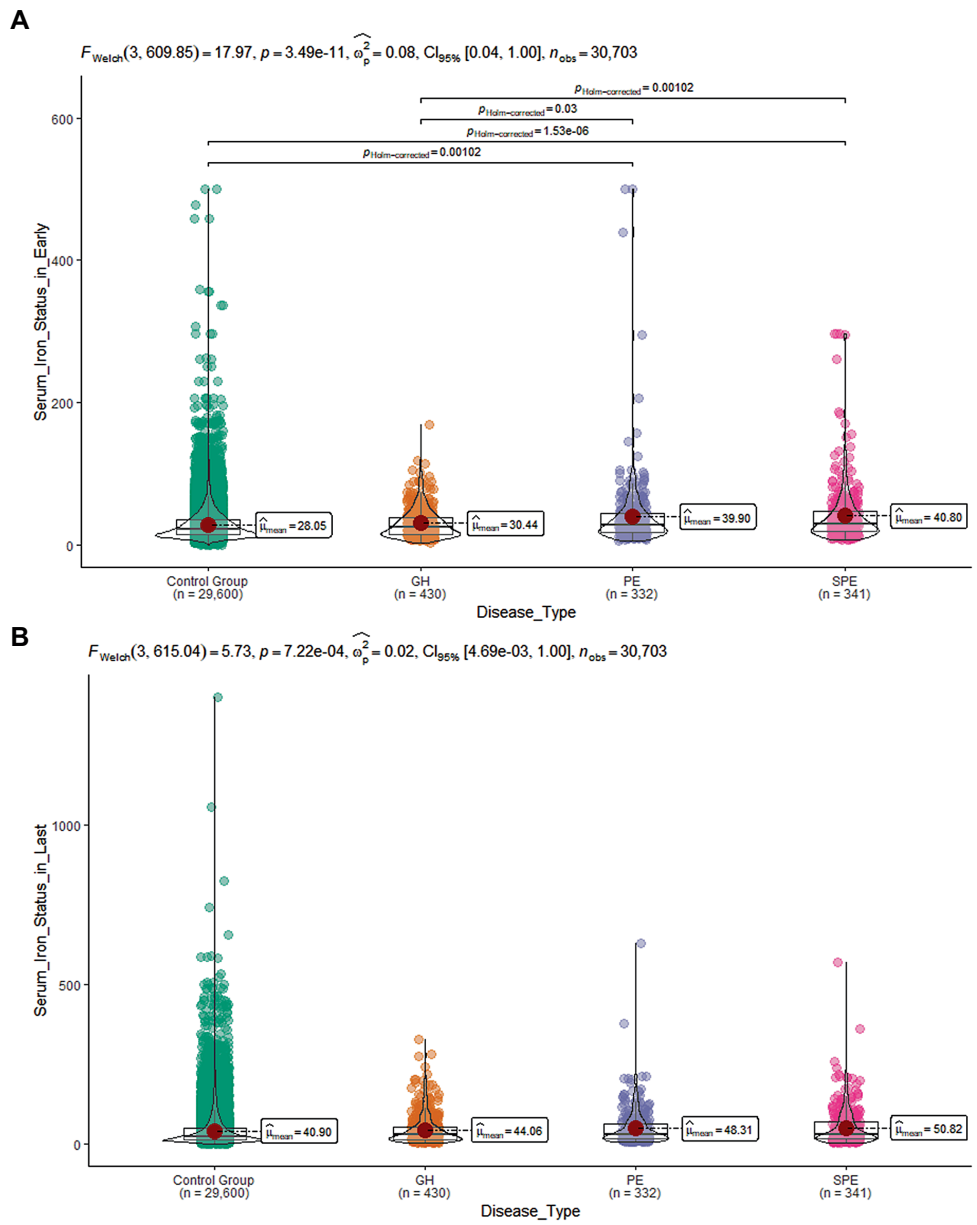
**(A)** SF levels during early pregnancy in the four group; **(B)** SF levels during late pregnancy in the four group.

Further two-by-two comparison revealed that SF increased with the severity of the disease (Figure 3). Since some of the baseline information differed between women with and without HDP, logistic regression models were constructed with HDP as the dependent variable and early pregnancy SF as the independent variable. The model was adjusted for confounders to estimate the adjusted odds ratio (AOR) for the occurrence of HDP in women with elevated SF in early pregnancy. After screening for confounding factors based on the results of univariate analysis and clinical significance (*p* < 0.1, i.e., univariate significance), age at delivery, pre-pregnancy BMI, net weight per week, ALT, ALB, GGT, AST, LDH, WBC, NE, TG, MO, G, P, HBV infection, GDM, thalassemia, hyperthyroidism, hypothyroidism, uterine malformation were gradually incorporated into the model. After correcting for relevant confounders (Model A-C), it was found that as the serum iron state in early pregnancy increased, so did the risk of HDP during pregnancy ((AOR) = 1.07, 95% CI [1.05,1.09]) (Table 2).

**Table 2:** Adjusted ORs for HDP according to serum ferritin in early pregnancy.

	Model A		Model B		Model C	
	AOR(95%CI)	*P value*	AOR(95%CI)	*P value*	AOR(95%CI)	*P value*
Serum ferritin in Early pregnancy	1.10[1.08,1.11]	<0.001	1.07[1.05,1.09]	<0.001	1.07[1.05,1.09]	<0.001
Age at delivery	1.03[1.02,1.05]	<0.001	1.02[1.01,1.04]	0.004	1.05[1.03,1.06]	<0.001
Pre-pregnancy BMI	1.20[1.18,1.22]	<0.001	1.20[1.18,1.22]	<0.001	1.20[1.18,1.22]	<0.001
Get weight per week	22.18[13.89,35.37]	<0.001	16.61[10.28,26.76]	<0.001	17.37[10.69,28.13]	<0.001
ALT	-	-	1.00[0.99,1.00]	0.083	1.00[0.99,1.00]	0.106
ALB	-	-	0.86[0.84,0.88]	<0.001	0.86[0.84,0.88]	<0.001
GGT	-	-	1.01[1.01,1.02]	<0.001	1.01[1.01,1.02]	<0.001
AST	-	-	1.02[1.01,1.03]	<0.001	1.02[1.01,1.03]	<0.001
LDH	-	-	1.00[1.00,1.00]	<0.001	1.00[1.00,1.00]	<0.001
WBC	-	-	1.05[1.02,1.07]	<0.001	1.05[1.02,1.07]	<0.001
NE	-	-	1.01[0.98,1.03]	0.540	1.00[0.98,1.03]	0.744
TG	-	-	1.17[1.13,1.22]	<0.001	1.16[1.11,1.20]	<0.001
MO	-	-	0.82[0.64,1.06]	0.134	0.84[0.65,1.08]	0.179
G	-	-	-	-	0.84[0.73,0.96]	0.011
P	-	-	-	-	0.66[0.57,0.76]	<0.001
HBV infection	-	-	-	-	0.86[0.70,1.05]	0.149
GDM	-	-	-	-	1.29[1.11,1.50]	0.001
Thalassemia	-	-	-	-	0.93[0.68,1.25]	0.635
Hyperthyroidism	-	-	-	-	1.56[1.03,2.26]	0.026
Hypothyroidism	-	-	-	-	1.02[0.74,1.36]	0.916
Uterinemalformation	-	-	-	-	1.19[0.62,2.07]	0.577

Model A: adjustment was made for Serum ferritin in Early (10 unit increments), Age at delivery, pre-pregnancy BMI, Get weight per week.

Model B: AOR were additionally adjusted for made for ALT, ALB, GGT, AST, LDH, WBC, NE, TG, MO.

Model C: adjustment was made for G, P, HBV infection, GDM, Thalassemia, Hyperthyroidism, Hypothyroidism, Uterine malformation in addition to the covariates in model B.

HDP, Hypertension Disorders in Pregnancy; AOR, adjusted odds ratio; ALT, Alanine aminotransaminase; AST, Aspartame aminotransferase; GGT, transglutaminase; ALB, Albumin; TG, Triglycerides; LDH, Lactate dehydrogenase; WBC, White blood cell count; NE, Neutrophil count; MO, Monocyte count.

### Early pregnancy SF thresholds for iron supplementation therapy

**Figure 4:**
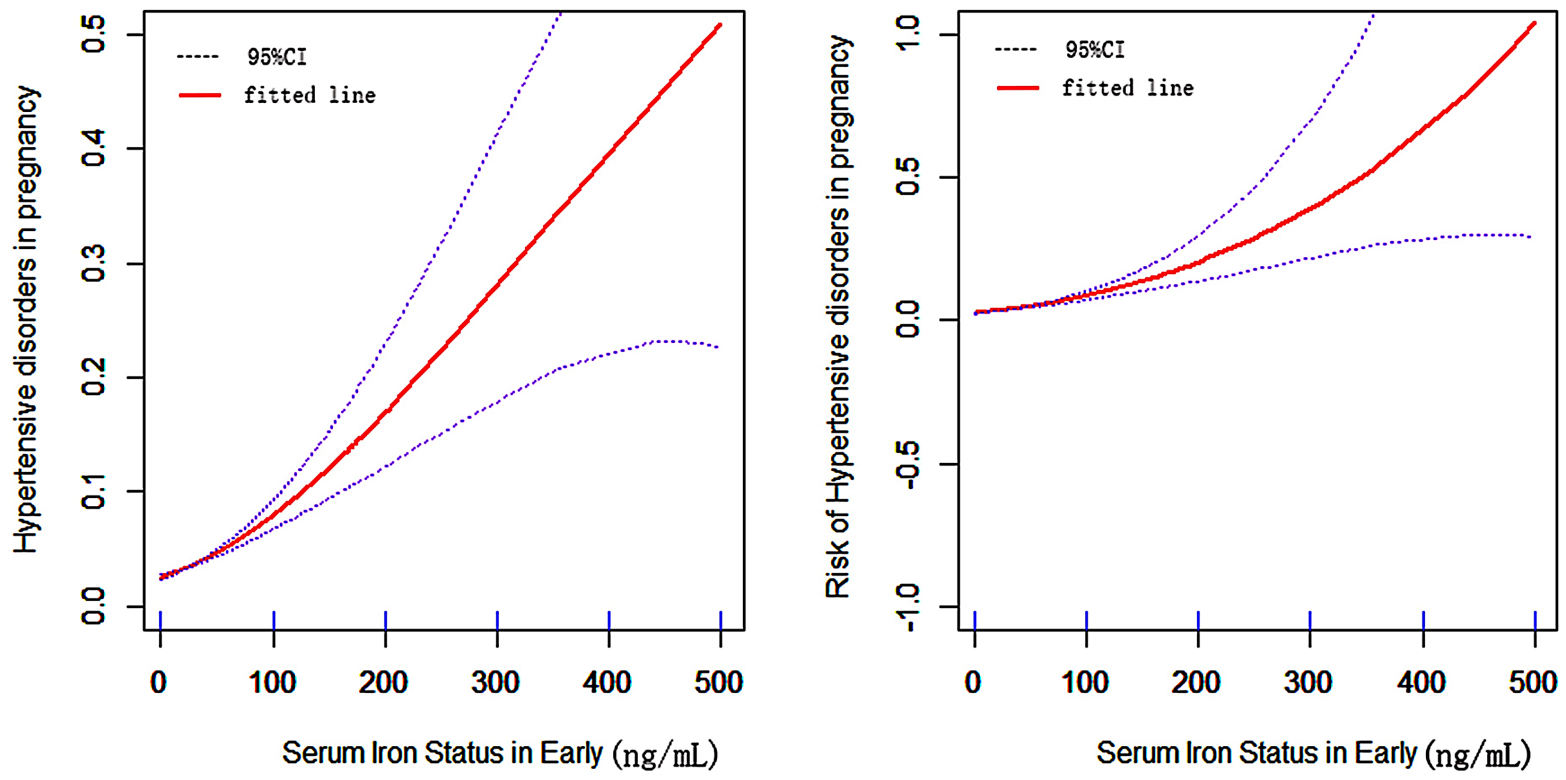
The relationship between SF in early pregnancy and hypertensive disorder in pregnancy. (Horizontal coordinates of both graphs are SF in early pregnancy, vertical coordinates of the left graph are the probability of developing HDP, vertical coordinates of the right graph are the risk of developing HDP, the solid line in the middle is the fitted line and the dashed lines on either side are 95% confidence intervals).

The risk of HDP increased relatively steadily with increasing SF in early pregnancy, with an inflection point around 70 ng/ml (Figure 4). There was a rapid increase in the risk of HDP after the inflection point. Further analysis of the threshold effect led to an inflection point of 64.22 mg/l.

## Discussion

We aimed to investigate the link between the levels of serum ferritin in early pregnancy and the development of pregnancy-related hypertensive disorders in a cohort of pregnant women who delivered in Fujian Provincial Maternal and Child Health Hospital. The results showed that early pregnancy SF was an independent risk factor for HDP. We showed that there is a threshold effect for the HDP risk with elevated SF.

In China, the prevalence of HDP is in the 5 to 10% range. HDP is among the top three causes of maternal death in China, responsible for 10.4% of maternal mortality in 2017 (12). Current preventive measures, recommended by the World Health Organization for preeclampsia include supplementing high-risk women with low-dose aspirin (13).

Recent 2020 study showed that around 19.8% of Chinese women are diagnosed with anemia and 13.9% had iron deficient anemia during pregnancy that becomes more prevalent over the progression of gestation (14).

SF level is often measured in women with anemia (15), and is considered an important indicator of the body’s iron reserves. Studies showed that serum iron and SF levels were significantly higher in pregnant women with preeclampsia compared to normal pregnancies, and that preeclampsia was liked to iron-induced oxidative stress (16). Similarly, Fenzl et al. explored micronutrient characteristics during pregnancy and found significantly increased levels of iron in the preeclampsia group (17). Furthermore, as summarized in a review by Erlandsson et al., iron is not only involved in the onset of preeclampsia but is also strongly associated with the severity of the disease (18). However, previous studies did not investigate the correlation of SF levels with the severity of the disease. In our study, hypertensive disorders in pregnancy were subdivided into a gestational hypertension, preeclampsia, and preeclampsia with severe features groups and investigated the correlation of early SF levels with the risk of developing each of the specific HDPs. Early pregnancy SF levels remained an independent risk factor for hypertensive disorders in pregnancy. Our conclusions confirm the results of the meta-analysis by Song et al. that showed an association between high serum iron levels and higher risk of HDP (6).

While iron is an factor that mediates oxidative stress (19), ferritin levels are indicative of the extent of systemic inflammation cellular damage as a result of the oxidative stress (20). Reactive oxygen species (ROS) and catalytic a4mounts of transition metal ions in ischemic tissue lead to generation of highly reactive hydroxyl radicals, through the Fenton reaction, a condition that contributes directly and indirectly to the process of oxidative stress. In recent years, ferroptosis has become a hot topic of research, and many scholars believe that ferroptosis-mediated abnormalities in oxidative stress may be involved in the pathogenesis of preeclampsia (21–23). The rapid increase in oxygen and iron during pregnancy leads to excessive membrane lipid peroxidation and ferroptosis in trophoblast cells at the maternal-fetal junction, resulting in reduced trophoblast infiltration capacity and impaired spiral artery recasting, which may contribute significantly to the development of hypertensive disorders in pregnancy.

Routine iron supplementation for women during pregnancy is commonly used to prevent iron deficiency anemia that is diagnosed based on the SF levels, with levels <30 micrograms/L indicative of the condition (24). However, studies show that the excessive iron levels correlate with higher risk of preeclampsia (25). Our study also shows that the risk of HDP increases with the increasing SF levels. Balancing iron supplementation therapy with the risk of HDP is, therefore, a current priority and challenge. In countries with scarce health resources, iron prophylaxis (i.e., iron supplements) in pregnancy is an effective preventive measure for all women. While this general form of iron supplementation therapy is effective in preventing iron deficiency anemia, it lacks some specificity as it can increase the risk of HDP in women with high levels of SF. Therefore, in developed countries with adequate health resources, iron supplementation should be administered to women who have a clear need for additional iron based on the SF concentrations (26). Current Chinese guidelines recommend the use of SF as a reference for iron supplementation therapy during pregnancy, with a cutoff levels of <30 mg/l (27). Our study found that early pregnancy SF was an independent risk factor for HDP, and this increased risk of HDP was more significant when early pregnancy SF was >64.22 mg/l. Therefore, early pregnancy SF can be used as a reference for iron supplementation therapy in pregnancy, and iron supplementation should not be routinely recommended for women with early pregnancy SF over 64.22 mg/l. Interestingly, we found that the association of early pregnancy SF levels with HDP was more significant than late pregnancy ferritin levels. Further studies are needed to find a mechanism of this difference.

The current study has some limitations. The main limitation of our study is its retrospective design, which introduces a risk of selection bias. The study refers to the Chinese population. Therefore, extrapolations of our results to other populations should be done with caution. We did not investigate the anemia status and iron supplementation in early pregnancy. While the contribution of these two factors to the correlation between serum ferritin in early pregnancy and HDP is relatively small, there is a chance that the lack of data on the anemia status and iron supplementation in our cohort may still impact our results. Additionally, although we observed a threshold effect between SF and hypertensive disorders in pregnancy, this threshold for the risk of developing HDR remains to be validated with larger sample sizes from multi-center studies.

## Conclusion

Our results show that high SF levels in early pregnancy are associated with the increased risk of HDP and identify specific threshold SF levels that justify iron supplementation. Our study has clear practical relevance for clinicians: early pregnancy SF levels may provide guidance for iron supplementation during pregnancy to balance the advantages and disadvantages of iron supplementation in terms of the risk of developing HDP.

## Data availability statement

The raw data supporting the conclusions of this article will be made available by the authors, without undue reservation.

## Ethics statement

The studies involving human participants were reviewed and approved by Ethical committee of the Fujian Maternity and Child Health Hospital approved this study (2020YJ183). The ethics committee waived the requirement of written informed consent for participation.

## Author contributions

ZF, SZ, YX, SL, HZ, and JY took part in substantial contributions to the study. ZF and SZ were the main contributors in the conceptualization of the work and involved in writing—original draft. YX and SL participated in the collection and analysis of clinical data. HZ and JY involved in review and editing. All authors had full access to all the data in the study and had final responsibility for the decision to submit for publication. All authors read and approved the final manuscript.

## Funding

This work was supported by the Fujian Natural Science Fundation Project (2022 J01425 and 2022 J011042), Joint Funds for the Innovation of Science and Technology, Fujian Province (2020Y9134), and Health Research Project of Department of Finance [Fujian finance refers to (2019) No. 827] (2020Y183). The funding sources had no role in the study design, data collection, data analysis, data interpretation, or preparation of the manuscript.

## Conflict of interest

The authors declare that the research was conducted in the absence of any commercial or financial relationships that could be construed as a potential conflict of interest.

## Publisher’s note

All claims expressed in this article are solely those of the authors and do not necessarily represent those of their affiliated organizations, or those of the publisher, the editors and the reviewers. Any product that may be evaluated in this article, or claim that may be made by its manufacturer, is not guaranteed or endorsed by the publisher.
